# Fundamental Limits to Position Determination by Concentration Gradients

**DOI:** 10.1371/journal.pcbi.0030078

**Published:** 2007-04-27

**Authors:** Filipe Tostevin, Pieter Rein ten Wolde, Martin Howard

**Affiliations:** 1 Department of Mathematics, Imperial College London, London, United Kingdom; 2 FOM Institute for Atomic and Molecular Physics (AMOLF), Amsterdam, The Netherlands; Lawrence Berkeley National Laboratory, United States of America

## Abstract

Position determination in biological systems is often achieved through protein concentration gradients. Measuring the local concentration of such a protein with a spatially varying distribution allows the measurement of position within the system. For these systems to work effectively, position determination must be robust to noise. Here, we calculate fundamental limits to the precision of position determination by concentration gradients due to unavoidable biochemical noise perturbing the gradients. We focus on gradient proteins with first-order reaction kinetics. Systems of this type have been experimentally characterised in both developmental and cell biology settings. For a single gradient we show that, through time-averaging, great precision potentially can be achieved even with very low protein copy numbers. As a second example, we investigate the ability of a system with oppositely directed gradients to find its centre. With this mechanism, positional precision close to the centre improves more slowly with increasing averaging time, and so longer averaging times or higher copy numbers are required for high precision. For both single and double gradients, we demonstrate the existence of optimal length scales for the gradients for which precision is maximized, as well as analyze how precision depends on the size of the concentration-measuring apparatus. These results provide fundamental constraints on the positional precision supplied by concentration gradients in various contexts, including both in developmental biology and also within a single cell.

## Author Summary

Many biological systems require precise positional information to function correctly. Examples include positioning of the site of cell division and determination of cell fate during embryonic development. This positional information often is encoded in concentration gradients. A specific protein is produced only within a small region, and subsequently spreads into the surrounding space. This leads to a spatial concentration gradient, with the highest protein concentration near the source. By switching on a signal only where the local concentration is above a certain threshold, this gradient can provide positional information. However, intrinsic randomness in biochemical reactions will lead to unavoidable fluctuations in the concentration profile, which in turn will lead to fluctuations in the identified position. We therefore investigated how precisely a noisy concentration gradient can specify positional information. We found that time-averaging of concentration measurements potentially allows for great precision to be achieved even with remarkably low protein copy numbers. We applied our results to a number of examples in both cell and developmental biology, including positioning of the site of cell division in bacteria and yeast, as well as gene expression in the developing *Drosophila* embryo.

## Introduction

To determine position in a biological system, some component within the system must have a nonuniform spatial distribution. Often, this is achieved through the formation of gradients of protein concentration. Typically, a gradient forms when a protein is manufactured/injected within a small region and subsequently spreads and decays [1]. By measuring the local concentration, position relative to the source can be determined. In developmental biology, where such gradients are used to control patterns of gene expression, gradient proteins are called morphogens. However, intracellular concentration gradients are also thought to be important for organisation inside single cells.

For a gradient mechanism to be biologically viable, position determination must be precise and therefore robust to noise. Variability from one copy of the system to another (e.g., from cell to cell or embryo to embryo) will certainly compromise positional precision. Production and degradation rates can vary (e.g., due to different copy numbers of transcription factors or proteases). The physical size of the system will also vary, and this may affect proper positioning. Most previous analyses of morphogen gradients have focused on robustness to changes in these extrinsic factors [2–4] between different copies of the system. However, there will also be intrinsic noise affecting the gradient within a single copy of the system, for example due to the unavoidably noisy nature of the biochemical reactions involved. This dissection of the fluctuations into extrinsic or intrinsic components mirrors that introduced into the analysis of stochastic gene expression [5–7]. However, here, intrinsic noise alters not only the overall protein copy numbers (similar to [5]), but also crucially the spatiotemporal protein distribution. Even if all extrinsic variation could be eliminated, intrinsic biochemical noise would still lead to a fundamental limit to the precision of position determination, in a similar way to limits on the precision of protein concentration measurement [8,9]. In this paper, we therefore address the question of how precisely a concentration gradient can specify positional information, and calculate the limits on positional precision for a simple, but biologically relevant, gradient formation mechanism with first-order reaction kinetics.

Quantitative measurements, for example on the Bicoid–Hunchback system in *Drosophila* [10], have shown that remarkable positional precision can sometimes be obtained. For this reason, understanding the fundamental limits to the precision of concentration gradients is clearly an important issue in developmental biology. Our results will be equally relevant to gradients that form within single cells, where protein copy numbers of a few thousand [11–13] will lead to large density fluctuations. The properties of intracellular protein gradients have been studied by Brown and Kholodenko [14]. Recently, a number of these gradients have been observed experimentally in both prokaryotic and eukaryotic systems. The bacterial virulence factor IcsA forms a polar gradient on the cell membrane of Shigella flexneri [15]. MipZ in Caulobacter crescentus forms polar gradients to aid division site selection [11]. In *Bacillus subtilis,* the MinCD complex also forms polar gradients in order to direct division site selection to the mid-plane of the cell [16,17]. In *Escherichia coli,* the oscillatory dynamics of the Min proteins creates a time-averaged gradient that directs cell division placement [18–24]. Using mechanisms of this sort, division site placement in bacteria can achieve an impressive precision of ±1% of the cell length [25,26]. Cell division in eukaryotic cells is also believed to be regulated by concentration gradients. For example, in fission yeast, the protein Pom1p forms a cortical concentration gradient emanating from a cell tip, thereby restricting the cell division protein Mid1p to the cell centre [27,28]. In eukaryotic cells, gradients of the Ran and HURP proteins aid the formation of the mitotic spindle by biasing microtubule growth toward the chromosomes [29–33]. Gradients may also play a role in the localization of Cdc42 activation, thereby permitting a coupling between cell shape and protein activation [34,35].

Suppose that a biological system needs to identify a particular position along its length, such as the mid-plane to ensure symmetrical cell division. As concrete examples, MipZ and the MinCD complex act by displacing the essential cell division protein FtsZ from the cell membrane. Since the concentrations of MipZ/MinCD are higher near the cell poles, FtsZ accumulates near the cell centre. Below some critical threshold of MinCD or MipZ concentration, enough FtsZ will presumably accumulate to form the division apparatus. The locations where the concentration gradient crosses these thresholds mark positions within the cell. In our analysis, we simply postulate the existence of such well-defined critical thresholds, where the gradient sharply switches a downstream signal from on to off. Clearly, any real gradient cannot act as such a sharp switch—in reality, a certain amount of smearing is inevitable. Furthermore, there will be additional noise in the process of actually measuring the concentration due both to the binding of the gradient proteins to the receptor molecules [8,9], and also to the downstream reactions that process this incoming signal [5–7,36–38]. In general, the noise of the output signal of a processing network can be written as the sum of a contribution from the noise in the input signal plus a contribution from the reactions that constitute the processing network. We assume here that the detector and the processing network are ideal and do not add any noise to the gradient input signal. As a result, our calculated variation constitutes a lower bound; any real gradient-signalling system will inevitably have a lower precision.

We first considered a system with a single planar morphogen source and linear degradation, thereby producing an exponentially decaying average concentration profile. While this model is very simple, it remains biologically relevant in both developmental and intracellular contexts. Gradients of Bicoid in *Drosophila* and IcsA in *Shigella* have been quantitatively measured and shown to fit this exponential decay profile on average to high accuracy [10,15]. We then calculated the expected distribution of positions where a noisy gradient crosses a concentration threshold. With typical cellular copy numbers of a few thousand proteins, the system would be unable to identify the correct threshold position from a single measurement. To achieve reliable position determination, the concentration must be averaged over time. We show that by averaging measurements, a biological system is able to achieve precision in position determination of a few percent of the system size even with hundreds of protein copies, a result we verified with computer simulations. Furthermore, we find that the precision of position determination is maximised when a particular choice of the gradient decay length is made. We also show how the precision depends on the detector size (i.e., the volume over which the density measurement is made). For a 2-D gradient (e.g., on a membrane), the precision possible after a certain averaging time depends only very weakly on the detector size. We relate all these results to experimental measurements of gradients in *Shigella* and fission yeast.

We also considered the ability of gradients from two poles to identify the centre of the system, as in the MipZ and Pom1p gradients discussed above. Related designs have also been proposed for the control of *hunchback* positioning in *Drosophila* [3,4,39]. As before, we find that the precision of the system can be optimised by a particular choice of the decay length. However, if the threshold position is set at the system centre, time-averaging improves precision more slowly than in the single-source model. For subcellular gradients, we find that a few thousand copies of the gradient proteins may therefore be required for high precision. Our results strongly constrain the possible concentrations of gradient proteins in two gradient systems.

## Results

### Single-Gradient Model

We considered a protein gradient that is used to determine a particular position along the length of a cylindrical system. The system will have dimension *d* = 2 if the gradient is restricted to the membrane, or *d* = 3 if the gradient is in the cytoplasm. We chose the *x*-axis along the long axis of the system. Position in the remaining coordinates is denoted by the vector **y**. For a membrane system, periodic boundary conditions are appropriate in the *y* direction. Otherwise, zero-flux boundaries are used throughout. The system length is *L*, and the size of the system in the remaining directions is taken to be *L*_⊥_ (so *L*_⊥_ = 2*πr*, where *r* is the system radius, for the *d* = 2 membrane case). A source on the *x* = 0 plane produces proteins at rate *J* per unit area, which then diffuse with diffusion constant *D*, and are degraded uniformly at rate *μ*. Neglecting fluctuations, the protein concentration *ρ*(*x*,**y**,*t*) is described by

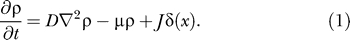
If *L ≫ λ* = (*D*/*μ*)^1/2^, the characteristic decay length of the gradient, we find that, at steady state, the density is

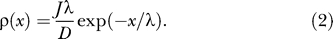
Symmetry dictates that the average density is independent of **y**. Gradients with the form of Equation 2 have been found to accurately fit quantitatively measured concentration profiles in both developmental [10] and subcellular [15] systems.

While we have outlined the model in terms of production and degradation, Equation 1 could equally apply to other mechanisms in which the active protein originates in a single location, but deactivation occurs uniformly throughout the system. The same equation would therefore describe a protein that is phosphorylated by a polar-localised kinase and dephosphorylated by a uniformly distributed phosphatase, or a protein that is activated by being injected into the membrane at a pole and deactivated when it dissociates. These biochemical details do not affect the behaviour of the model.

We suppose that signalling is active where the local gradient protein concentration is above some threshold value, *ρ_T_*, and inactive otherwise. The average concentration profile for a single gradient, Equation 2, suggests that the system will be divided into a region 0 ≤ *x* < *x_T_* where signalling is active, and a region *x_T_* ≤ *x* ≤ *L* where signalling is not active, with *ρ_T_* = *ρ*(*x_T_*). However, noise in the local protein concentration will cause this threshold position to fluctuate. This noise may come from intrinsic fluctuations in the diffusion, injection, and decay processes, or from extrinsic factors that produce systematic changes in the boundary position when comparing one copy of the system to another. Here we consider only intrinsic biochemical fluctuations.

Protein production and degradation events were considered to be single-molecule reactions with a fixed probability per unit time, and hence were Poisson processes. We also assumed that the hopping of proteins in or out of a particular region of space is governed by Poisson statistics, thereby generating a diffusive process for molecular transport. Since the system is linear, the instantaneous fluctuations in molecular number, *n*, within a volume (Δ*x*)*^d^* centred on the position (*x*,**y**) should also obey Poisson statistics, with

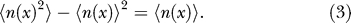
In terms of protein density, this becomes

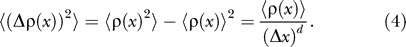
This relation can also be established using more elaborate field theoretic techniques (see [40]). From this expression for the variation in the density, we can compute the width of the threshold position distribution by expanding the average threshold position *x_T_*. To leading order, this width is given by

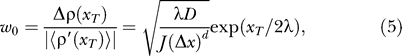
where *ρ*′(*x_T_*) denotes the first derivative of the density at *x* = *x_T_*.

Here we identify (Δ*x*)*^d^* as the size of the region in which the concentration is being measured. For subcellular gradients involved in positional information, this volume is determined by the size of an individual receptor or protein with which the gradient protein interacts, an example being the interaction between the MinCD and FtsZ proteins in *B. subtilis.* The size of the detector, Δ*x*, will then be on a molecular scale. This conclusion still holds even if the gradient proteins bind cooperatively to the “detection” protein/receptor due to the close physical proximity of the bound molecules. In contrast, however, the cellular length scale will be much larger, 1μm or bigger.

Throughout the following analysis we focus on subcellular gradients. However, our model can equally be applied to developmental biology, and we consider these systems further in the Discussion. As concrete examples, we first consider the IcsA polar gradient on the membrane of the rod-shaped bacterium *Shigella* (*L* ≈ 3 μm, *L*_⊥_ ≈ 3 μm) [15]. IcsA is exported to the outer membrane at a single pole, after which it diffuses and undergoes uniform proteolysis by the protease IcsP, thereby forming an exponential gradient exactly as in our model [15]. Outer membrane IcsA is then able to recruit actin nucleation factors. However, a critical concentration of IcsA is likely needed for actin nucleation: in this way a comet-like actin tail is generated at only one cell pole, thereby generating unidirectional motility of the pathogen. A cell will typically have a few thousand copies of IcsA [12], forming a gradient with *λ* ≈ 0.5 μm [15]. We take the detector size to be Δ*x* = 0.01 μm, consistent with an interaction between IcsA and actin nucleation proteins. For diffusion on the cell membrane, we take *D* = 1 μm^2^s^−1^. On the membrane of a cell of this size, there would be approximately *LL*_⊥_/(Δ*x*)^2^ ∼ 10^5^ potential detector sites, many more than the typical copy number. Even near to the source pole, detector sites will typically be unoccupied. A detector region at a distance *x* = 0.5 μm from the highly occupied pole will have average occupancy of <*n*> ∼ 10^−1^. In the cytoplasm of a similarly sized bacterium, the number of potential detector sites will be ∼10^6^, again much larger than the protein copy numbers typically supported by bacteria.

Similar estimates can be made for single polar gradients in fission yeast (*L* = 10 μm, *L*_⊥_ = 6 μm), such as for Pom1p [27,28]. Here we assume a total of 2,000 protein copies (this concentration has not yet been measured but this number is plausible [28]). We also take *D* = 1 μm^2^s^−1^ and a decay length of *λ* = 2 μm, parameters that are approximately consistent with the Pom1p gradient imaged by Padte et al. [28]. We again assume that Δ*x* = 0.01 μm, corresponding to a molecular-sized detector, as would be the case if the gradient protein interacted with other membrane proteins (such as Mid1p) [27,28]. The typical occupancy of a Δ*x* = 0.01 μm site is then <*n*> ∼ 10^−2^ at *x* = 2 μm from the source.

As we have seen for both fission yeast and *Shigella,* average detector site occupancies that are very much less than one protein per site ensure that the threshold occupancy must necessarily be less than one. Since most regions will be devoid of any copies of the protein, a single instantaneous measurement of the protein density cannot give a good estimate of the local average concentration. In addition, multiple positions where the concentration crosses *ρ_T_* would be observed simultaneously in such a measurement since the concentration would be above the threshold everywhere there is a protein molecule present, and below the threshold where there is no protein molecule. To reliably determine the average concentration profile, the system must therefore integrate the measured concentration over time.

The noisy concentration profile provided by the gradient protein forms the input signal that is then time-averaged by a downstream signal-processing network. In general, the mechanism for time-averaging is provided by the lifetimes of the states in the processing network. For instance, in the case of gene expression, fluctuations in the occupancy of the promoter by a gene regulatory protein can be filtered by the lifetime of the mRNA transcript, provided that lifetime is much longer than the timescale of fluctuations in the promoter occupancy [7,9]. Similarly, for subcellular gradients, as in *Shigella,* fluctuations in the gradient can be filtered by the lifetime of activated receptors/detector proteins or their downstream products. Provided this timescale is much longer than the sub-millisecond timescale of the gradient fluctuations, good time-averaging can then be achieved. Importantly, the reactions in the downstream network not only time-average the noise of the input signal, but also add further noise to the signal [5–7,36–38]. Here, we focus exclusively on noise in the concentration gradient and do not model the downstream reactions explicitly, but simply assume they are noiseless and model them with an effective averaging time. In essence, we assume that the detector and the network that process the gradient signal are ideal and do not add further noise, and are thus able to time-average the gradient signal in the best possible way. Our results thus provide a lower bound to the output noise set by the Poissonian fluctuations of the signalling molecules.

We suppose that averaging over a time interval *τ* we can take *N_τ_* = *τ*/*τ_ind_* independent measurements of the concentration. In our ideal case, we then expect that the fluctuations in the concentration would decrease according to *N_τ_* ^−1/2^. Since the width varies linearly with Δ*ρ* according to Equation 5, the width will also decrease as

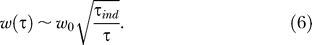
The timescale *τ_ind_* on which independent measurements can be made is set in our ideal case solely by the reaction–diffusion dynamics of the gradient proteins, as discussed in Methods. For cellular parameter values, the typical reaction timescale, 1/*μ*, will be much longer than the typical timescale for diffusion between detector sites, (Δ*x*)^2^/*D*. Assuming a molecular-sized detector, this latter timescale would be on the order of 10^−4^ s, whereas effective protein lifetimes will typically be seconds or longer. The Damkohler number for the system, the ratio of the diffusive and reaction timescales, would therefore be *Da* ∼ (Δ*x)* ^2^/*λ* ^2^ ∼ 10^−4^. Since *Da* ≪ 1, the averaging timescale is dominated by diffusive motion. In *d* = 3 we find *τ_ind_* ∼ (Δ*x*)^2^/*D*. However, in *d* = 2, density correlations decay away more slowly, leading to the appearance of logarithmic corrections that are weakly dependent on the parameters *λ* and Δ*x*. For long averaging times, *τ* ≫ 1/*μ*, the width determined from time-averaged measurements would be

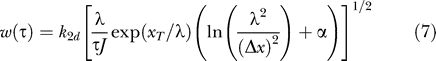
in *d* = 2, and for *d* = 3

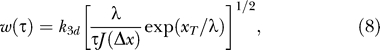
where *k*_2*d*_, *k*_3*d*_, and *α* are constants.

As we have discussed above, Δ*x* will be set by the concentration detection mechanism. However, in a subcellular context, Δ*x* also sets the highest possible resolution of the system. Once *w* ≈ Δ*x*, the cell cannot resolve the target position with any higher precision. Equation 7 suggests that in *d* = 2, precision depends only very weakly on the detector size, through the logarithmic correction factor. Reducing the detector size would increase the number of independent measurements made in a given averaging time. However, since fewer proteins would be measured by each detector over one averaging period, reducing Δ*x* would therefore increase the instantaneous density fluctuations. In *d* = 2, these two effects largely cancel. Hence, even if we have over/underestimated the detector volume, this will have little effect on the precision of gradients in *d* = 2 dimensions, such as IcsA in *Shigella* or Pom1p in fission yeast. In three dimensions, however, *w* varies as (Δ*x*)^−1/2^. Since increasing Δ*x* reduces *w* in both *d* = 2 and *d* = 3, an optimal strategy would be to choose Δ*x* to match the desired precision in order to minimise the required averaging time.

Intriguingly, from Equations 7 and 8 we find that there exists an optimal decay length such that precision is maximised. This result can be understood as follows: for fixed *x_T_*, and for *λ* ≫ *x_T_*, the value of |<*ρ*′(*x_T_*)>| tends to a constant *J*/*D*, independent of *x_T_*. However, as *λ* increases, <*ρ*(*x_T_*)> increases and therefore the absolute size of the fluctuations in the density also increases. Therefore, for large and increasing values of *λ*, *w* ∝ <*ρ*(*x_T_*)^1/2^> / |<*ρ*′(*x_T_*)>| must be increasing. Now if *λ* is smaller than *x_T_* and decreasing, when computing the width ∝ <*ρ*(*x_T_*)^1/2^> / |<*ρ*′(*x_T_*)>|, the presence of the square root means that the numerator decreases much more slowly than the denominator. Hence, the width must again increase as *λ* is decreased for small *λ.* Combining these results for small and large *λ*, the width must have a minimum, optimum value as a function of *λ.* This occurs at *λ* = *x_T_* in *d* = 3. In *d* = 2, the optimal decay length is given approximately by

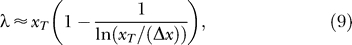
in which we have retained the first-order logarithmic correction.

**Figure 1:**
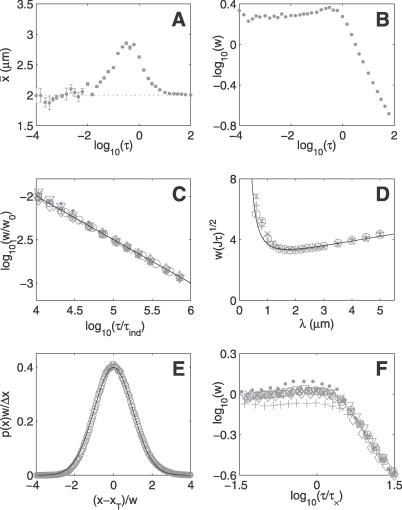
Single-Gradient Model in *d* = 2 (A) Variation of the estimated threshold position with averaging time, with *x_T_* = 2 μm and *λ* = 2 μm. (B) Variation of the width as a function of averaging time. (C) Data collapse of the width at large *τ* for a range of parameter values. Full line shows the prediction of Equation 7 with *k*_2*d*_ = 0.40 and *α* = 2.5. (D) *w*(*τ*) as a function of decay length, with *x_T_* = 2 μm. Results for three different averaging times are shown: ×, *τ* = 10 s; circle, *τ* = 15 s; and +, *τ* = 22.5 s. The full line shows the prediction from Equation 7. At large *λ*, the simulation results deviate from the prediction since the assumption that *L* ≫ *λ* is no longer valid. (E) Plot of the probability distribution for measuring the threshold at position *x* with an averaging time *τ* = 45 s. The full line shows a normal distribution. (F) Scaling of the crossover time, *τ_×_*, according to Equation 13. In (A), (B), and (E), the standard parameter values given in the text were used. In (C) and (F), * indicates the standard parameter values. For the other datasets, one parameter value was changed as follows: open circle, *D* = 0.5 μm^2^s^−1^; open square, *J* = 6.25 μm^−1^s^−1^; ×, Δ*x* = 0.02 μm; closed circle, *μ* = 1 s^−1^; +, *μ* = 0.11 s^−1^; open diamond, *x_T_* = 1 μm; and inverted triangle, *x_T_* = 3 μm.

To examine the biological impact of Equation 7 we again considered the Pom1p membrane gradient in fission yeast [27,28] using the parameters described earlier. Simulations of this example system were performed as described in Methods, with on average 100 proteins in the system. Figures 1A and 1B show how the measured threshold position, *x̄*, and width, *w*, vary with averaging time. For long averaging times, the simulation data gives excellent agreement with Equation 7, with the constants *k*_2*d*_ = 0.40 ± 0.02 and *α* = 2.5 ± 0.8. Figure 1C shows the *w* ∼ *τ* ^−1/2^ behaviour predicted in Equation 7, and Figure 1D confirms that the width has a minimum as a function of *λ*. The simulation results are consistent with the position of the minimum predicted by Equation 9. Figure 1E shows that the distribution of measured threshold positions is Gaussian to a good approximation.

Since the averaging timescale *τ_ind_* in a subcellular system is on the order of ∼10^−4^ s, time-averaging over a period of minutes can achieve great precision even with very few copies of the gradient protein. With the parameter values given above, Equation 7 predicts that the position *x_T_* = 2 μm can be located to within ±0.5 μm within an averaging time *τ* = 60 s even if the system contains on average only about 20 copies of the protein. A precision of ±0.1 μm can be achieved in the same averaging time with about 400 copies of the protein, a remarkably high level of precision for such a low concentration. In vivo Pom1p gradients may be formed by a few thousand protein copies, allowing for even greater precision.

However, we can see in Figure 1B that for averaging times of less than about a second, the simulation results are not consistent with Equation 7. In this regime both *w* and *x̄*; are equal to *λ*. As discussed above, at very short averaging times the presence of a particle at any position will cause the time-averaged concentration to be above *ρ_T_* at that point and hence generally will generate a threshold crossing. The probability distribution of threshold measurements, *p*(*x*), will therefore follow the probability distribution of particles. Assuming *L* ≫ *λ*, we have

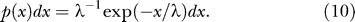
The cell will on average estimate the threshold position to be

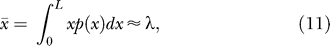
and measurements will be distributed about this position with variance

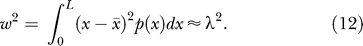
The system is therefore unable to resolve the correct threshold position at these short timescales if this is different from *λ.*

Associated with the average concentration at the threshold is a length scale, *l* ∼ *ρ_T_* ^−1/*d*^, the typical distance between proteins at this position. The average time for a protein to diffuse this distance will scale as *l^2^* / *D*. In two dimensions, this time is given by

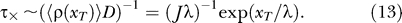
Since *τ_×_* is the timescale on which a diffusing particle first arrives at *x_T_*, if *τ ≪ τ_×_*, there will generally be no particles detected at *x_T_* in the averaging period. The system therefore cannot reliably estimate the mean concentration at *x_T_*, and hence cannot precisely identify the threshold position. For averaging times much greater than *τ_×_*, on average at least one particle will be detected at *x_T_*. The time-averaged concentration profile will then approach Equation 2, and *x̄* will approach *x_T_*. Hence *τ_×_* determines the crossover time between the two observed regimes of constant *w* and *w* ∝ *τ* ^−1/2^. Figure 1F shows that the scaling in Equation 13 is also reproduced in our simulations. For the parameter values above, *τ_×_* = 0.3 s, and for a more realistic copy number of 1,000, *τ_×_* = 0.03 s. These timescales are extremely short compared with cell-cycle timescales, but do nevertheless show that some sort of time-averaging is probably essential: a single instantaneous measurement is unlikely to provide precise positional information. In fact, as we have seen, averaging over much longer times (tens of seconds) may be necessary if very high (1%) precision is required.

Simulations of the model in *d* = 3 were also performed (unpublished data). Similar behaviour was observed in this case, and Equation 8 gave good agreement with the observed width at long averaging times.

### Oppositely Directed Gradients

To reliably locate the centre of a system, the mechanism responsible must incorporate information about the overall system size so that the identified position can scale correctly. A single gradient characterised by a fixed decay length cannot achieve this. We therefore examined a system where protein gradients are produced by sources at both ends, and where the central position is identified as a concentration minimum.

We modified our earlier model by adding an additional planar source at *x* = *L*. This addition is appropriate for modelling cell division inhibitors, such as MipZ in *Caulobacter,* that are injected into the membrane near both cell poles. However, our model would apply equally if the two sources were of different repressor proteins (as may be the case in fission yeast [27,28]), although we do assume that *J*, *D*, and *μ* are the same for both gradients. In this scenario, signalling activity would be determined by the total concentration. Without fluctuations, this would be described by

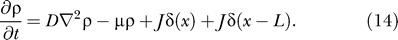
The steady-state solution is now

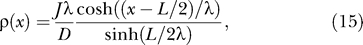
which has the expected minimum at *x* = *L*/2.

We then supposed that the cell compares the concentration to a threshold value corresponding to the minimum of the average profile, *ρ_min_* = *ρ*(*L*/2) = *ρ_T_*. Positions where the concentration is at or below the threshold are identified as being at the centre of the cell. While the average steady-state density profile would never extend below *ρ_min_*, fluctuations ensure that the concentration in the region around the centre spends a significant amount of time at or below the threshold. Around point(s) where <*ρ*(*x*)> = *ρ_T_*, noise in the protein concentration would lead to a distribution of threshold-crossing positions. We considered an expansion of the density fluctuations about *x_T_* = *L*/2, giving, to leading order

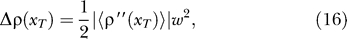
since any first-order term proportional to <*ρ*′> vanishes at *x_T_* = *L*/2. The width is therefore given by

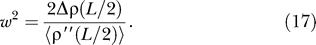
Substituting in Equation 15 gives

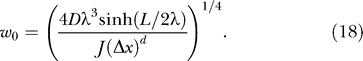



As in the single-gradient model, the typical occupancy of the threshold region would be much less than one. For example, if we take the parameter values considered previously for the Pom1p gradient in fission yeast, with 2,000 protein copies, the average occupancy of a detector site at *x* = *L*/2 would be <*n*(*L*/2)> ∼ 10^−3^. We assume here that Pom1p forms a gradient from both poles. In fact, it may only form a single gradient, with another hitherto unidentified protein forming the second polar gradient [27,28]. However, as discussed earlier, this detail does not affect our calculations. As a second example, MipZ in *Caulobacter* (*L* = 2.5 μm, *L*_⊥_ = 2 μm) is typically present at about 1,000 copies, and forms two polar gradients with a decay length *λ* ≈ 0.25 μm [11]. The average occupancy at the centre of this system would be approximately <*n*(*L*/2)> ∼ 10^−3^. Averaging measurements of the concentration over time is therefore required in both cases to obtain precise positional information. Since the width now goes as (Δ*ρ*)^1/2^, as shown in Equation 17, we expect

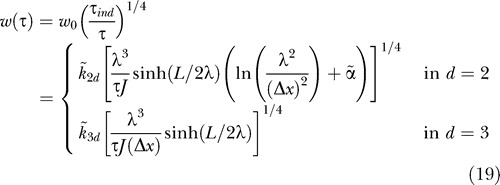
where 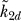 , 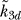 , and Α~ are constants. Averaging proceeds much more slowly than previously, with a *τ* ^−1/4^ dependence. This follows directly from the vanishing of the first derivative at the average threshold position. In *d* = 3, and for *λ* ≪ L, Equation 19 predicts that *w* will be minimised when *λ* ≈ *L*/6 is chosen. In *d* = 2, logarithmic corrections again alter this result slightly, with the optimal decay length now occurring at

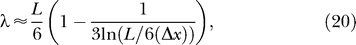
in which we have included the leading logarithmic correction. This optimal length scale arises for similar reasons as in the single-gradient model. For the Pom1p gradient imaged by Padte et al [28], the decay length is observed to be 1–1.5 μm, comparable with this optimal decay length of about 1.5 μm for a 10-μm cell.

**Figure 2:**
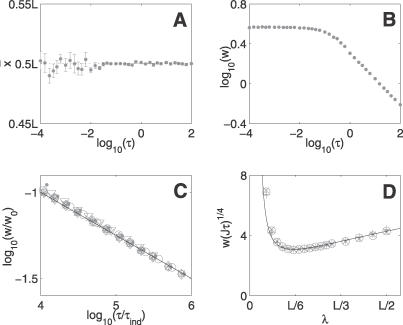
Two-Gradient Model in *d* = 2 (A) The mean threshold position fluctuates about *L*/2 due to the symmetry of the system. (B) Variation of the width *w* as a function of averaging time. (C) Data collapse of the width as a function of averaging time, at long times, for a range of parameter values. The full line shows Equation 19 with *k~*_2*d*_ = 0.63 and Α~ = 2.5. * indicates the standard parameter values. For the other datasets, parameter values were changed as follows: open circle, *D* = 0.5 μm^2^s^−1^; open square, *J* = 9 μm^−1^s^−1^; ×, Δ*x* = 0.02 μm; closed circle, *μ* = 1 s^−1^; +, *μ* = 0.25 s^−1^; diamond, *L* = 7.5 μm; and inverted triangle, *L* = 15 μm and Δ*x* = 0.02 μm. (D) Plot of width as a function of decay length for averaging times: ×, *τ* = 30 s; open circle, *τ* = 45 s; and +, *τ* = 60 s. The full line shows the prediction from Equation 19.

We simulated our model in *d* = 2 with representative parameter values for fission yeast membrane gradients. We used *μ* = 0.36 s^−1^, chosen to give *λ* = 1.67 μm, and *J* = 6 μm^−1^s^−1^, giving, on average, 200 protein copies in total. Figure 2 shows the results of these simulations. Again, we observe two distinct regimes. At averaging times longer than about a second, there is excellent agreement with Equation 19, as we can see in Figure 2C. Fitting to the simulation results, we find 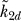 = 0.63 ± 0.02 and Α~ = 2.5 ± 1.0. Figure 2D confirms the existence of the optimal decay length in our simulations.

Since the width decays as *τ* ^−1/4^ for this system, longer averaging times and/or higher protein copy numbers are required than in the single-gradient model to achieve high precision. Intrinsic biochemical noise may therefore strongly constrain systems of this type. For the yeast-membrane gradient considered above to achieve a precision of ±5% of the cell length after averaging for 1 min, about 800 protein copies are required. Therefore, in the absence of any other positioning mechanisms, the Pom1p gradient will require ∼1,000 protein copies or more to precisely direct the location of cell division. We estimate that the MipZ gradient in *Caulobacter,* with 1,000 protein copies, would be able to locate the cell centre to within ±5% of *L* after approximately *τ* = 2 s. However, since precision only improves as *τ* ^−1/4^, averaging over *τ* = 20 min would be required for the same system to achieve ±1% accuracy.

## Discussion

Noise in biochemical processes within a cell will lead to fluctuations in protein concentration gradients, and hence also to variation in the position where these gradients cross a particular threshold value. These fluctuations therefore place a limit on the potential precision of position determination mechanisms relying on concentration gradients alone. In subcellular systems with protein copy numbers in the thousands, this noise will be sufficiently large that position cannot be determined reliably from a single measurement of the density profile. To determine position to within a few percent of the system length, a precision achieved by some subcellular systems, the protein concentration must be averaged over time. For a single subcellular membrane gradient, we have seen that by averaging over a period of a minute, excellent precision can potentially be achieved with only a few hundred protein copies. This remarkable precision is due to the sub-millisecond diffusive timescale on which time-averaging occurs. Precise identification of the cell mid-plane by gradients emanating from both poles requires longer averaging times or higher copy numbers, since larger fluctuations result from the vanishing first derivative of the average concentration at the system centre. Intrinsic biochemical noise may therefore be a strong constraint on subcellular two-gradient positioning systems, dictating that the copy numbers be sufficiently high to suppress fluctuations.

So far we have focused almost exclusively on fluctuations in subcellular gradients; however, our results are also applicable to developmental biology, and we wish to comment briefly on this application. Here, the appropriate length scales are usually much longer, on the order of hundreds of micrometers in *Drosophila.* Moreover, the gradients affect patterns of gene expression through the binding of gradient molecules to DNA regulatory sequences inside individual nuclei. For example, in *Drosophila,* where exponential gradients have been quantitatively measured for Bicoid [10], Bicoid binds cooperatively to *hunchback* regulatory DNA. In this case we again expect molecular-scale effective measuring volumes, with Δ*x* ∼0.01 μm as a reasonable order of magnitude. We next assume purely Poisson statistics for the fluctuations: this is a stronger assumption than for our earlier subcellular gradients, as there will be additional complications arising, for example, from the import/export of morphogens from nuclear compartments. However, if diffusive noise is dominant, then Poisson statistics would be retained, and we could expect our earlier analysis to apply, although with one important distinction. Instead of Δ*x* setting the maximal possible precision, this would now be set by the size of individual nuclei (prior to cellularization), since we expect relatively homogeneous gene expression within a single nuclear volume. A single nucleus in *Drosophila* has a length scale of about 10 μm, still much smaller than the decay length of the gradient of *λ* ∼100 μm, allowing for high-precision gene expression [10]. Using the *Drosophila* Bicoid gradient as an example, we use *L* = 500 μm, *L*_⊥_ = 100 μm, and estimate *D* = 10 μm^2^s^−1^ and *μ* = 10^−3^s^−1^, giving *λ* = 100 μm, consistent with experimental measurements [10]. Assuming a high copy number of 10^7^ per embryo (we are not aware of experimental constraints on this figure) gives *J* ∼ 1 μm^−2^s^−1^. For a single gradient in *d* = 3, we find that about a 5-min averaging time is required to bring the error down to ±1 nuclear length. For a two-gradient model in *d* = 3, longer averaging times on the order of an hour are required to reduce the centre-finding positional error to about ±2 nuclear lengths. Since gene expression may need to be controlled on shorter timescales than this, other designs (e.g., using *interacting* gradients [3,4]) may be required for high-precision centre-finding (see also below). The effects of the optimum gradient length scale will also be interesting to probe in a developmental biology context. However, our simple analysis may be complicated by the multiple roles played by many morphogens: for example, Bicoid not only activates *hunchback,* but it also helps to regulate pair-rule genes, such as *Even-skipped.* Nevertheless, it is interesting to note that the Bicoid gradient length scale *λ* ∼ 100 μm [10] is not too far away from the *L*/6 optimum for a two-gradient system, and in a single-gradient context will offer maximal precision well into the anterior half of the embryo.

Up to this point we have only considered systems with first-order degradation. Morphogen gradients with nonlinear decay have also been proposed [2]. This nonlinearity will lead to non-Poissonian density fluctuations, which may significantly change the observed behaviour. England and Cardy [41] have previously calculated the response of a gradient with nonlinear decay to one source of biochemical noise, namely a fluctuating production rate. However, they calculated the change to the average gradient, while fluctuations about this average may also be important. It would certainly be of interest to compare the performance of linear and nonlinear degradation mechanisms in more detail. Centre-finding mechanisms with interactions have also been proposed [3,4]. In these models, position is determined from the combined gradient of two proteins, which would be steep around the system centre due to an interaction between the two gradients. These mechanisms may therefore be able to achieve greater precision for midpoint determination than the noninteracting mechanism considered here.

Throughout this work we have assumed that the gradient protein concentration fluctuates about a steady-state profile, and hence averaging over a longer time will give a more precise estimate of the average profile. For a subcellular system, the steady-state gradient will develop over timescales of less than about a minute, due to the micrometer length scales involved. This timescale is short compared with the cell-cycle time, which ranges from tens of minutes up to many hours. For this reason we expect that subcellular gradients will be in steady state, and therefore that our analysis will be directly applicable. However, in developmental biology, the effective lifetimes will likely be much longer, and the gradient may take hours to fully reach steady state. Moreover, a number of developmental biology systems are known to respond to a morphogen gradient that has not reached steady state [42–44]. A further complication is the possibility of gradient formation by non-Fickian diffusion [45], where there is no steady state at all. The model considered in this paper does not take into account time-varying average gradients. If the average gradient is evolving, a longer averaging period will not necessarily lead to improved precision. Clearly, more work will be required to understand how such dynamically evolving systems are able to yield precise positional information and filter out fluctuations. Nevertheless, we do note that two-gradient systems of the kind analyzed here are naturally able to locate the system centre even without being in steady state, due to the symmetry of the system [3]. The positional variations in such a non–steady-state scenario will not be the same as calculated here, but our analysis does form a first step toward the analysis of these more complex systems.

## Methods

### Calculation of *τ_ind_.*

We have assumed in our analysis that during the time-averaging process we are taking independent measurements at intervals of *τ_ind_*. However, in both real biological systems and our simulations, measurements can generally be taken at much shorter intervals than this, leading to correlations between consecutive measurements. For a series of correlated measurements taken at time intervals *δt* over a period 0 ≤ *t* ≤ *τ*, with *τ* ≫ *δt*, the expected error for the time-averaged concentration at position **x**, (Δ*ρ*(**x**,*τ*))^2^, is given by [46]

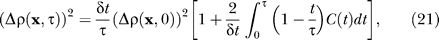
where (Δ*ρ*(**x**,0))^2^ is the variance of a single measurement,

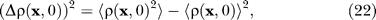
and *C*(*t*) is the normalized density correlation function,

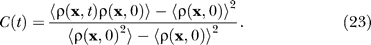
We therefore define the timescale *τ_ind_* to be

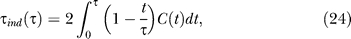
and assuming *τ_ind_* ≫ *δt*, we recover

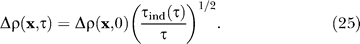
For *N* independent measurements of the density, we would expect the error to decline as *N* ^–1/2^. For large enough values of *τ_ind_*(*τ*), where *τ_ind_* becomes independent of *τ*, we can therefore interpret *τ_ind_* as the time interval required for successive measurements to be independent.

The next step of the calculation is to compute the correlation function *C*(*t*) appropriate for our model. For pure diffusion, we expect:

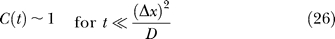


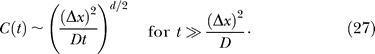
On timescales *t* ≪ (Δ*x*)^2^/*D*, the system remains perfectly correlated, as there has been insufficient time for particles to hop away to neighbouring sites. However, for *t* ≫ (Δ*x*)^2^/*D*, an algebraically decaying correlation function is found, characteristic of diffusion. However, we also need to incorporate the effects of spontaneous decay that occur independently of the diffusive motion. Adding decay to the system simply alters the correlation functions by a multiplicative factor of exp(−*μt*). We now substitute this full form into the definition of *τ_ind_* (Equation 24). In the biologically relevant limits where *τ* ≫ (Δ*x*)^2^/*D* and 1/*μ* ≫ (Δ*x*)^2^/*D*, we find, for *d* = 2

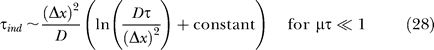


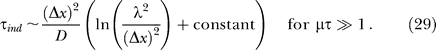
In *d* = 3, we find

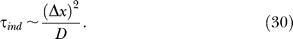
For the parameter values considered in our simulations, we do not observe the logarithmic *τ* dependence in the width predicted by Equation 28. In the single-gradient simulations, this is because at short times *τ* ≪ *τ*_×_, we enter the constant *w* ∼ *λ* regime. For the parameter values used, the transition from *w* ∼ *λ* at *τ* ≪ *τ_×_* ≈ 0.3 s to the long-time behaviour (Equation 7) for *τ* ≫ 1/*μ* ≈ 4 s overwhelms the small logarithmic effect. If the production rate *J* were increased significantly, *τ_×_* ∝ *J* ^−1^ would be reduced and the ln(*τ*) regime would become accessible since the *τ_×_* and 1/*μ* timescales would then become better separated. However, even in this case, the logarithmic variation in Equation 28 is intrinsically weak, and would likely have a negligible effect in a biological context.

### Simulations.

Stochastic simulations were performed on a 2-D square lattice with *N_x_* = *L*/*δx* sites in the *x* direction and *N_y_* = *L*_⊥_/*δx* sites in the *y* direction, where *δx* = 0.01 μm is the lattice spacing. The detector size Δ*x* was normally set equal to *δx* except for cases where the detector size was varied, in which case Δ*x* was set to be a multiple of *δx*. Zero-flux boundaries were implemented at *x* = 0 and *x* = *L*, and a periodic boundary was used to connect *y* = 0 with *y* = *L*_⊥_. A fixed time step, *δt* = 2.5 × 10^−5^ s, was chosen so that for the given diffusion constant the total probability of diffusion out of a site in all directions approached 1. However, a time step five times smaller was also tested with no effect on any of the results. For each *x* = 0 site, particles were injected at each time step in a Poisson process with mean *j* = *Jδxδt*. In the two-gradient model, particles were also added at *x* = *L* in an identical but uncorrelated process. Diffusion and decay were also treated as Poisson processes, with hopping and decay probabilities of *Dδt*/(*δx*)^2^ and *μδt* per particle, respectively. Simulations were initialised with the mean number of particles in the system, *JL*_⊥_/*μ* for the one-gradient model or twice this value for the two-gradient model, with a probability distribution that followed the average density distribution.

The mean occupancy for each detector site was calculated over the averaging period, *τ*. For each site this mean occupancy was compared with each neighbouring site. If one occupancy was above the threshold and the other below, this boundary was identified as a threshold-crossing position. This process was repeated for many averaging periods, ranging from 10^5^ repeats for short averaging times to 500 repeats for very long averaging times, to generate a distribution of crossing positions throughout the system. Threshold crossings in both the *x* and *y* directions were observed. We found that the distributions as a function of *x* position of these two types of crossing were the same. For each row of sites, *x* = 0 to *x* = *L* at a fixed *y*, the mean (“measured threshold”) and root-mean–squared deviation (“width”) of the threshold distribution from many averaging periods were calculated independently. In Figures 1 and 2, we plot the mean of these two quantities across the different *y* values within the system, with error bars of one standard deviation.

For the single-source model, the standard parameter values used in the simulations were as follows: *L* = 10 μm, *L*_⊥_ = 6 μm, *D* = 1 μm^2^s^−1^, *μ* = 0.25 s^−1^, *J* = 4.17 μm^−1^s^−1^, Δ*x* = 0.01 μm, and *x_T_* = 2 μm. To generate the data collapse in Figures 1C and 1F, simulations were also performed with the following parameter values: *D* = 0.5 μm^2^s^−1^; *J* = 6.25 μm^−1^s^−1^; Δ*x* = 0.02 μm; *μ* = 1 s^−1^; *μ* = 0.11 s^−1^; *x_T_* = 1 μm and *x_T_* = 3 μm. For the two-source model, standard parameters were the same as above except *μ* = 0.36 s^−1^ and *J* = 6 μm^−1^s^−1^. In Figure 2C, data are also shown with the following parameter values: *D* = 0.5 μm^2^s^−1^; *μ* = 1 s^−1^; *μ* = 0.25 s^−1^; *J* = 9 μm^−1^s^−1^; Δ*x* = 0.02 μm; *L* = 7.5 μm; *L* = 15 μm, and Δ*x* = 0.02 μm.
